# Phosphocholine – an agonist of metabotropic but not of ionotropic functions of α9-containing nicotinic acetylcholine receptors

**DOI:** 10.1038/srep28660

**Published:** 2016-06-28

**Authors:** K. Richter, V. Mathes, M. Fronius, M. Althaus, A. Hecker, G. Krasteva-Christ, W. Padberg, A. J. Hone, J. M. McIntosh, A. Zakrzewicz, V. Grau

**Affiliations:** 1 Laboratory of Experimental Surgery, Department of General and Thoracic Surgery, German Centre for Lung Research, Justus-Liebig-University Giessen, Giessen, Germany; 2 Department of Physiology, University of Otago, Dunedin, New Zealand; 3 Institute for Animal Physiology, Justus-Liebig-University of Giessen, Giessen, Germany; 4 Intitute for Anatomy and Cell Biology, Julius-Maximilians-University of Wuerzburg, Wuerzburg, Germany; 5 Department of Biology, University of Utah, Salt Lake City, Utah, USA; 6 George E. Wahlen Veterans Affairs Medical Center, Salt Lake City, Utah, USA; 7 Department of Psychiatry, University of Utah, Salt Lake City, Utah, USA

## Abstract

We demonstrated previously that phosphocholine and phosphocholine-modified macromolecules efficiently inhibit ATP-dependent release of interleukin-1β from human and murine monocytes by a mechanism involving nicotinic acetylcholine receptors (nAChR). Interleukin-1β is a potent pro-inflammatory cytokine of innate immunity that plays pivotal roles in host defence. Control of interleukin-1β release is vital as excessively high systemic levels cause life threatening inflammatory diseases. In spite of its structural similarity to acetylcholine, there are no other reports on interactions of phosphocholine with nAChR. In this study, we demonstrate that phosphocholine inhibits ion-channel function of ATP receptor P2X7 in monocytic cells via nAChR containing α9 and α10 subunits. In stark contrast to choline, phosphocholine does not evoke ion current responses in *Xenopus laevis* oocytes, which heterologously express functional homomeric nAChR composed of α9 subunits or heteromeric receptors containing α9 and α10 subunits. Preincubation of these oocytes with phosphocholine, however, attenuated choline-induced ion current changes, suggesting that phosphocholine may act as a silent agonist. We conclude that phophocholine activates immuno-modulatory nAChR expressed by monocytes but does not stimulate canonical ionotropic receptor functions.

Phosphocholine (PC) is a precursor as well as a degradation product of phosphatidylcholine (lecithin), a major phospholipid of eukaryotic bio-membranes^1^,^2^. Beside the important role of PC for eukaryotic bio-membranes, PC moieties can be covalently attached to proteins and glycolipids of plants^3^, fungi^4^, eukaryotic parasites^5^, and some pathogenic bacteria^6^,^7^. PC and PC-modified macromolecules interact with C-reactive protein (CRP), a classical acute phase reactant^8^,^9^, or with natural antibodies^7^,^10^. These interactions may induce complement fixation and other effector mechanisms involved in host defence. In contrast, PC-modified macromolecules are also known to provoke strong anti-inflammatory effects and contribute to immune evasion of parasites^5^,^11^. For example, PC-modified lipopolysaccharide (LPS) from *Haemophilus influenzae* contributes to bacterial virulence and enables persistent host colonization^6^,^12^,^13^. The mechanisms, regarding how PC-modified macromolecules initiate immune evasion, are not fully understood. PC-modifications on the surface of *H. influenzae* might be a kind of molecular mimicry preventing activation of *Toll-like* receptors (TLR)^14^,^15^. Interestingly, *Myd88*, an adaptor molecule involved in signalling of TLR and interleukin-1 (IL-1) seems to be of importance, as wild-type and PC-deficient *H. influenzae* strains are cleared at the same pace in *Myd88*-deficient mice^15^.

Two isoforms of the pro-inflammatory cytokine IL-1 exist, IL-1α and IL-1β, which signal via the same receptors and play a central role in host defence against infections^16^. IL-1α mainly acts locally within infected tissues, whereas IL-1β exerts more systemic effects^16^. High concentrations of circulating IL-1β, however, can cause life-threatening systemic inflammatory response syndrome (SIRS)^17^. Hence, IL-1β release is tightly controlled and the underlying mechanisms are of significant clinical interest. Production of mature IL-1β by human monocytes and macrophages typically depends on two consecutive danger signals such as LPS and extracellular ATP^18^,^19^. LPS activates TLR-4 and induces the biosynthesis of pro-IL-1β that remains within the cytoplasm, unless the cell becomes activated by another danger signal. Extracellular ATP originating from damaged neighbouring cells typically binds to ATP-receptor P2X7, induces efflux of K^+^ ions, assembly of the NLRP3 inflammasome, activation of the proteolytic activity of caspase-1, cleavage of pro-IL-1β, and release of mature bioactive IL-1β^18^,^19^.

Recently, we demonstrated that free PC and PC-modified macromolecules dose-dependently inhibit ATP-induced release of IL-1β by human monocytic cells^20^. In the same study we showed that a similar effect is provoked by nicotine, acetylcholine (ACh) and choline (Cho)^20^. The inhibitory effect of PC moieties is antagonized by mecamylamine (Mec), α-bungarotoxin and strychnine, suggesting that nicotinic acetylcholine receptors (nAChR) containing α9 and/or α10 subunits are involved in signalling^20^. Interestingly, nicotine abolishes ATP-induced ion channel functions of P2X7 receptors in U937 cells, a human monocytic cell line, but does not provoke ion currents itself 20.

The purpose of this study was to test the hypothesis that binding of PC and Cho to nAChR inhibit P2X7 receptor function similar to nicotine. In addition, we hypothesised that PC is a novel agonist of nAChR containing α9 and/or α10 subunits and directly compare the effects of PC to the well-known α9^*^ (* indicates the possible presence of additional nAChR subunits) agonist Cho^21^. We provide evidence that PC and Cho induce metabotropic effects via α9α10*-containing nAChR in monocytic cells that result in an inhibition of P2X7 receptor function. Canonical ionotropic functions of α9* nAChR, however, are triggered by Cho but strikingly not by PC.

## Results

### PC and Cho inhibit BzATP-induced IL-1β release via α9 and α10 nAChR subunits

**Figure 1:**
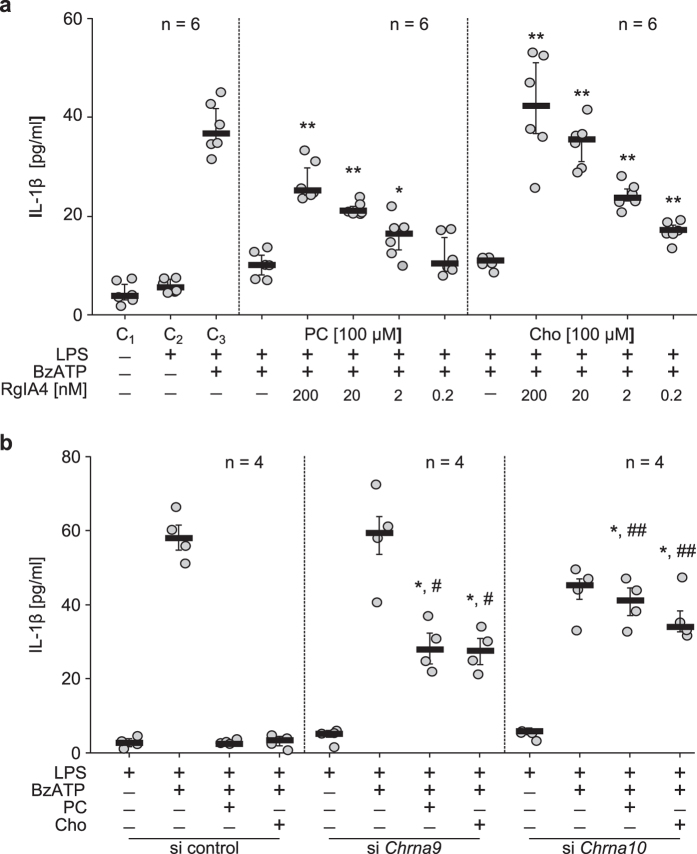
Choline and phosphocholine inhibit BzATP-induced IL-1β release from U937 cells via nicotinic acetylcholine receptor containing α9 and α10 subunits. (**a**) Human monocytic U937 cells were primed with lipopolysaccharide (LPS, 1 µg/ml, 5 h) followed by stimulation with BzATP (100 µM, 30 min) in the presence or absence of phosphocholine (PC, 100 µM), choline (Cho, 100 µM) and/or different concentrations of the α-conotoxin RgIA4, a specific antagonist of α9α10 nicotinic acetylcholine receptors (nAChR). Interleukin-1β (IL-1β) released into the culture medium was measured by enzyme linked immunosorbent assay (ELISA). In control experiments, cells were left untreated (C1), primed with LPS (C2) or with LPS followed by BzATP (C3). In the presence of PC as well as Cho the IL-1β release was inhibited. The inhibitory effect of PC and Cho was dose-dependently antagonized by RgIA4 (*P ≤ 0.05, **P ≤ 0.01, significantly different from cells treated with PC or Cho alone, Mann-Whitney rank-sum test). (**b**) In LPS-primed U937 cells that were transfected with control siRNA (si) the BzATP-stimulated IL-1β release was inhibited by PC and Cho. In cells transfected with siRNA to Chrna9 or Chrna10, the effects of PC and Cho were blunted (*P ≤ 0.05, different from cells treated with LPS and BzATP; ^#^P ≤ 0.05, ^##^P ≤ 0.01, different from respective experiments on cells treated with control siRNA; Kruskal-Wallis followed by Mann-Whitney rank-sum test). Data are presented as individual data points, bar represents median, whiskers percentiles 25 and 75.

To test the hypothesis, that the inhibitory effects of PC and Cho are mediated via nAChR containing α9 and/or α10 subunits, we used an analogue of α-conotoxin RgIA (RgIA4, synonym CSP-4), a potent and selective antagonist of human α9* nAChR^22^,^23^. Human monocytic U937 cells were primed with LPS for 5 h followed by stimulation with the selective P2X7 agonist BzATP (2′(3′)-O-(4-benzoyl-benzoyl)ATP trieethylammonium salt)^24^ for another 30 min in the presence or absence of PC, Cho and/or RgIA4. Thereafter, IL-1β released into the culture medium was measured by an enzyme linked immunosorbent assay (ELISA; Fig. 1). As expected, PC or Cho (100 μM each) completely inhibited BzATP-induced IL-1β release (Fig. 1a). RgIA4 fully antagonized the inhibitory effect of PC and Cho in a dose-dependent manner (IC_50_ about 10 nM; n = 6; P ≤ 0.05; Fig. 1a).

To corroborate these results, we transfected U937 cells with small interfering RNA (siRNA) to silence the expression of α9 and α10 nAChR subunits or with scrambled control siRNA. The efficiency of this treatment in U937 cells was shown before^20^. Transfection of control siRNA neither impaired BzATP-induced release of IL-1β nor altered the inhibitory effects of PC and Cho (n = 4; P ≤ 0.05; Fig. 1b). In contrast, when the expression of α9 or α10 subunits was silenced by siRNA the inhibitory effect of PC as well as Cho was blunted (n = 4; P ≤ 0.05; Fig. 1b).

**Figure 2:**
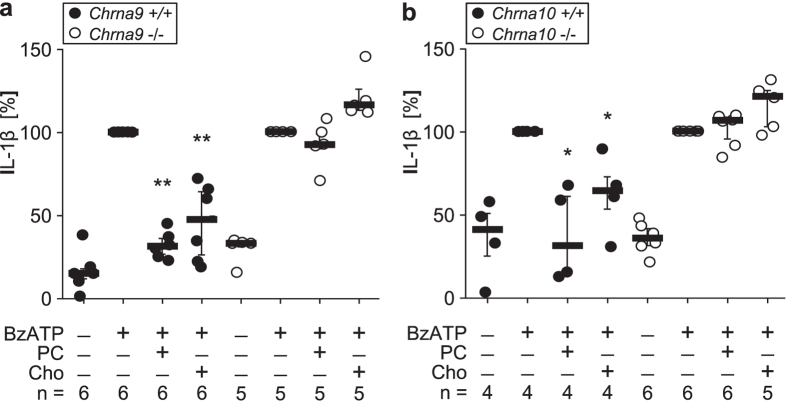
Choline and phosphocholine do not inhibit BzATP-induced IL-1β release from mononuclear leukocytes of Chrna9 and Chrna10 gene-deficient mice. (**a,b**) Mononuclear leukocytes were isolated from Chrna9 and Chrna10 gene-deficient mice (white circle; Chrna9 −/−; Chrna10 −/−) and corresponding wild-type mice (black circle; Chrna9 +/+; Chrna10 +/+). BzATP (100 µM) induced release of interleukin-1β (IL-1β) was investigated in the presence of phosphocholine (PC; 100 µM) or choline (Cho; 100 µM). PC and Cho suppressed BzATP-induced release of IL-1β in all WT strains investigated. In sharp contrast, no inhibition of IL-1β release was seen in Chrna9 −/− and Chrna10 −/− mice deficient in α9 or α10 subunit containing nicotinic acetylcholine receptors, suggesting that both subunits are needed (*P ≤ 0.05, **P ≤ 0.01, significantly different from cells treated with PC or Cho alone, Mann-Whitney rank-sum test). Data are presented as individual data points, bar represents median, whiskers percentiles 25 and 75.

Furthermore, we investigated BzATP-induced IL-1β release by peripheral blood mononuclear leukocytes (PBMC) isolated from *Chrna9* and from *Chrna10* gene-deficient mice (*Chrna9* −/−; *Chrna10* −/−) as well as from two corresponding wild-type (WT) strains (*Chrna9* +/+; *Chrna10* +/+). BzATP consistently induced release of IL-1β from WT and gene-deficient PBMC (n ≥ 4; Fig. 2). PC or Cho (100 μM each) significantly reduced BzATP-induced IL-1β release from PBMC isolated from *Chrna9* +/+ (n = 6; P ≤ 0.01) and *Chrna10* +/+ (n = 4; P ≤ 0.05) mouse strains (Fig. 2). In contrast, PC and Cho were ineffective in PBMC from *Chrna9* −/− or *Chrna10* −/− mice (n ≥ 5; P ≥ 0.05; Fig. 2).

At the end of each experiment, lactate dehydrogenase (LDH) levels were determined to test for cell viability. As shown in Table 1 and Table 2, LDH values remained below 10% of the total release, irrespective of the experiment performed.

### PC and Cho inhibit BzATP-induced ion current stimulation in U937 cells

**Figure 3:**
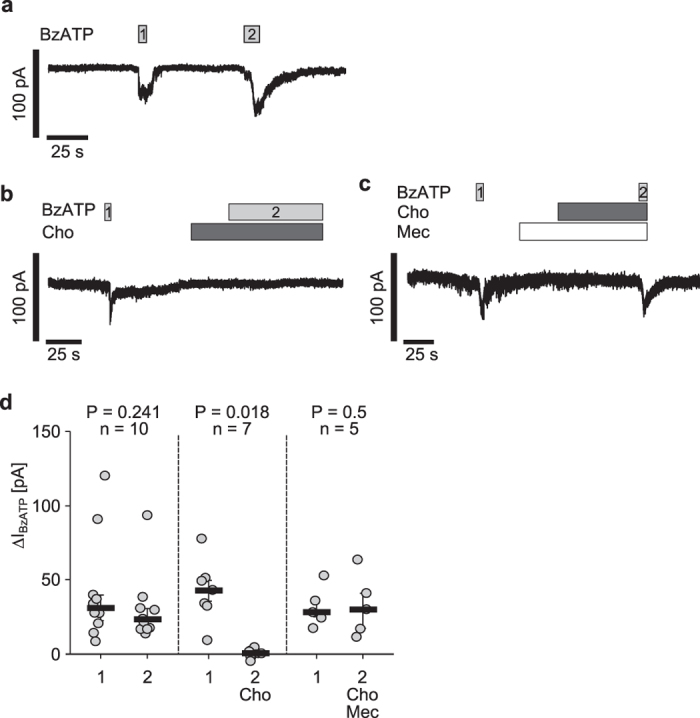
Choline inhibits BzATP-induced ion current stimulation in U937 cells. Whole-cell patch-clamp measurements were performed on human monocytic U937 cells primed with lipopolysaccharide (1 µg/ml, 5 h). Depicted are representative current curves (**a,c**). (**a,d**) In control experiments, consecutive application of the P2X7 receptor agonist BzATP (100 µM, grey bar) induced repetitive ion current stimulations (BzATP1 and 2). (**b,d**). After washout of the first BzATP stimulus, choline (Cho, 100 µM, dark grey bars) was applied. In presence of Cho, BzATP did not change the ion current. (**c,d**) Mecamylamine (Mec, 100 µM, white bar) antagonized the inhibitory effect of Cho. All BzATP-induced current changes (∆I_BzATP_) are shown as individual data points, bars represent median, whiskers percentiles 25 and 75. Statistical analyses were performed using the Wilcoxon signed-rank test.

To investigate if BzATP-induced ion current stimulation due to P2X7 receptor activation is inhibited by PC and Cho, we performed whole-cell patch-clamp measurements on LPS-primed U937 cells. As shown previously^20^, application of BzATP (100 μM) consistently induced ion currents (Fig. 3a,d). BzATP-induced ion current stimulation was reversible by washout and repeatable (Fig. 3a,d). No significant changes were detected when comparing the amplitude (∆I_BzATP_) of the first BzATP-induced response with the second (n = 10, P = 0.241; Fig. 3d), indicating that the receptors do not desensitise under these conditions. In the next set of experiments BzATP was first applied alone, which provoked ion currents (Fig. 3b). After washout, the cells were preincubated with Cho (100 μM) for 30 s, followed by an additional application of BzATP (Fig. 3b). Cho alone did not cause any changes in current (n = 7, Fig. 3b). Moreover, Cho abolished BzATP-induced current stimulation (n = 7, P = 0.018; Fig. 3b,d). In the next experiments, cells were preincubated with the nAChR antagonist Mec (100 μM), followed by application of Cho (Fig. 3c). Under these conditions (Mec + Cho) a BzATP-induced current stimulation was detectable and the first and second BzATP-induced effects did not differ (n = 5, P = 0.5; Fig. 3c,d).

**Figure 4:**
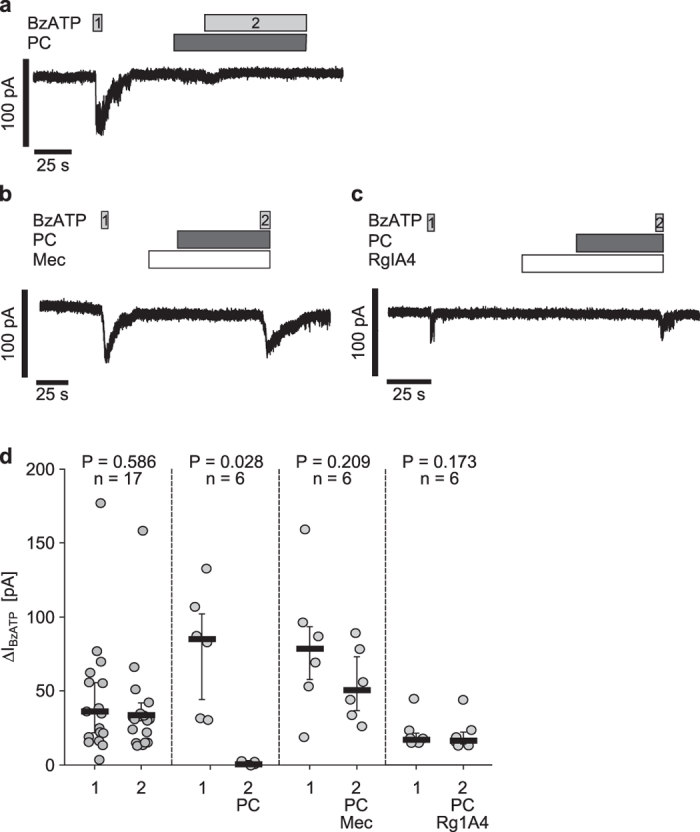
The inhibitory effect of phosphocholine on BzATP-mediated ion current stimulation is antagonized by α-conotoxin RgIA4. Whole-cell patch-clamp measurements were performed on human monocytic U937 cells primed with lipopolysaccharide (1 µg/ml, 5 h). (**a**) Application of the P2X7 receptor agonist BzATP (100 µM, grey bar) induced an ion current stimulation (BzATP1). After washout of BzATP1, phosphocholine (PC, 1 mM, dark grey bars) was applied. In presence of PC, BzATP did not change the ion current (BzATP2). (**b,c,d**) Mecamylamine (Mec, 100 µM, white bar, (**b**) as well as RgIA4 (200 nM, white bar; (**c**) antagonized the inhibitory effect of PC. (**d**) In parallel performed control experiments application of BzATP induced repetitive ion current stimulations that did not differ (current curve not shown). All ∆I_BzATP_ values are shown as individual data points, bars represent median, whiskers percentiles 25 and 75. Statistical analyses were performed using the Wilcoxon signed-rank test.

We performed the same kind of experiments using PC (1 mM) instead of Cho (Fig. 4). PC alone did not induce ion current changes. The BzATP-induced effect was abolished in presence of PC (n = 6, P = 0.028; Fig. 4a,d). Furthermore, the inhibitory effect of PC was antagonized by preincubation of the cells with Mec and the first and second BzATP-induced effect did not differ (n = 6, P = 0.209; Fig. 4b,d). Taken together, we were able to show that PC and Cho inhibit BzATP-induced ion fluxes in U937 cells.

Previous experiments examining the inhibition of BzATP-induced IL-1β release by PC provided evidence that nAChR containing subunits α9 and/or α10 are involved (Fig. 1). To confirm these data, we performed whole-cell patch-clamp measurements in the presence of RgIA4 (200 nM) (Fig. 4c). For this purpose, the BzATP-induced current was determined (n = 6; Fig. 4c). After washout of BzATP, cells were preincubated with RgIA4 and followed by PC application (Fig. 4c). Subsequent application of BzATP activated a current that was similar to the preceding BzATP current (n = 6, P = 0.173; Fig. 4c,d), suggesting that RgIA4 fully antagonized the inhibitory effect of PC.

### PC does not induce ion current stimulation in *Xenopus laevis* oocytes expressing α9 or α9α10 nAChR

Cho is an agonist of nAChR containing α9 subunits^21^. To test if PC also evokes ion currents at canonical α9 containing receptors, human α9 subunit as well as a combination of human α9 and α10 subunits were heterologously expressed in *Xenopus laevis* oocytes. Two-electrode voltage-clamp (TEVC) measurements were performed to assess ion channel functions of nAChR.

**Figure 5:**
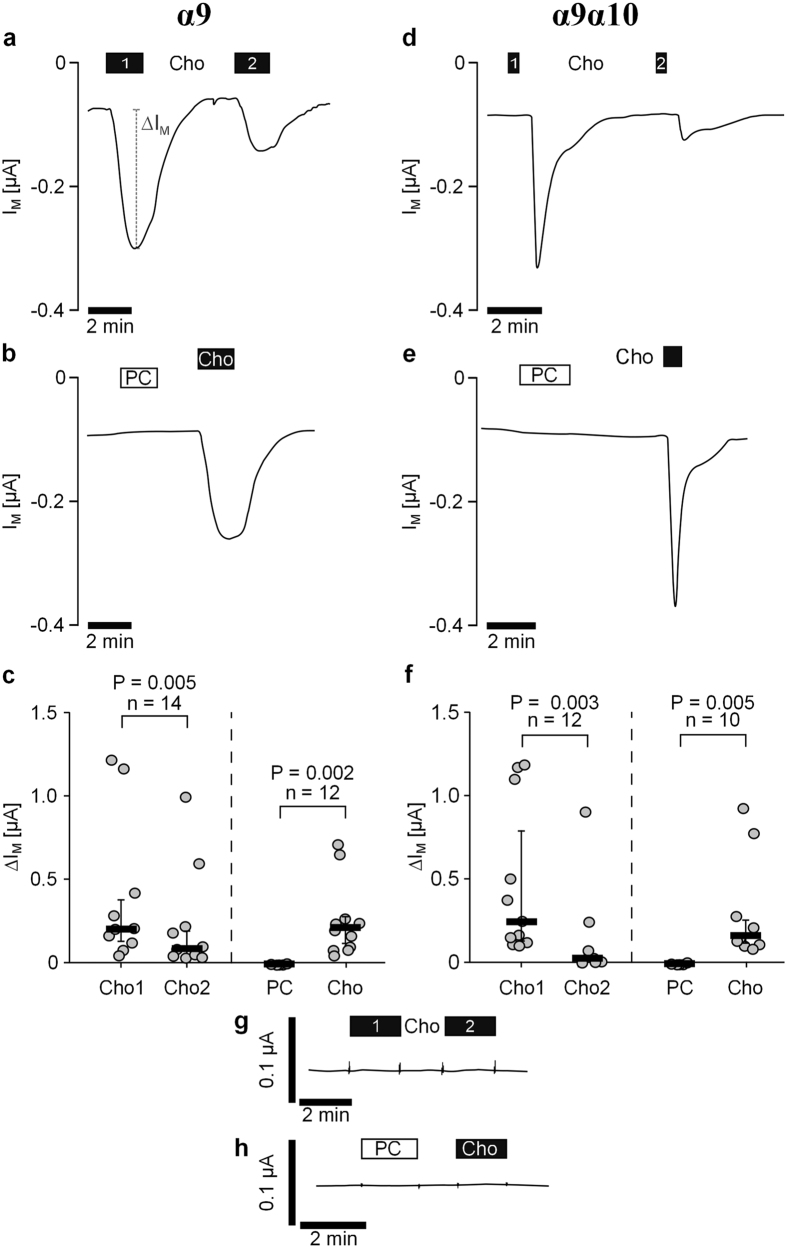
Phosphocholine does not induce ion channel functions in Xenopus laevis oocytes that heterologously express α9 or α9α10 nicotinic acetylcholine receptors. Two-electrode voltage-clamp (TEVC) measurements were performed on oocytes that heterologously expressed human α9 alone (**a–c**) or a combination of α9 and α10 (**d–f**) nicotinic acetylcholine receptor (nAChR) subunits. (**a,d**) Choline (Cho, 1 mM, black bars) induced repetitive stimulations of the transmembrane ion current (IM) in oocytes transfected with α9 (**a**) and in oocytes co-expressing α9α10 nAChR subunits (**d**). The second Cho-induced effect (Cho2) was smaller compared to the first one (Cho1) indicating receptor desensitization. (**b,e**) Initial application of phosphocholine (PC, 1 mM, white bars) had no impact on IM, whereas application of Cho thereafter induced a current stimulation. Again, oocytes expressing only α9 and those co-expressing α9α10 nAChR subunits led to similar results. (**g,h**) Representative current traces of water injected control oocytes (no expression of human receptors). Neither repeated application of Cho (n = 17), nor PC (n = 11) induced any changes in I_M_. Depicted are representative current curves (**a,b,d,e,g,h**). All changes of the transmembrane current (∆I_M_) induced by cholinergic stimulation are shown as individual data points, bars represent median, whiskers percentiles 25 and 75 (**c,f**). Statistical analyses were performed using the Wilcoxon signed-rank test.

In oocytes expressing homomeric α9 nAChR, application of Cho (Cho1) resulted in a rapid stimulation of the transmembrane current (I_M_; Fig. 5a,c). The effect of Cho was reversible upon washout (Fig. 5a). Subsequent application of Cho (Cho2) activated a current that was significantly smaller than the preceding Cho-induced current (n = 14, P = 0.005; Fig. 5a,c). In contrast, when PC was applied for 2 min, no ion currents were provoked (Fig. 5b). As an internal positive control, Cho was applied at the end of each experiment resulting in a significant current stimulation (n = 12, P = 0.002; Fig. 5c).

The same kinds of experiments were performed on oocytes co-expressing nAChR subunits α9 and α10. Application of Cho induced a current stimulation (Fig. 5d,f). In comparison to the Cho-induced effect in oocytes expressing α9 (Fig. 5a), the current stimulation was faster and shorter (Fig. 5d). The Cho effect was repeatable and significantly blunted compared to the first response (n = 12, P = 0.003; Fig. 5f). Again, application of PC had no impact on the current while Cho induced a current stimulation (n = 10, P = 0.005; Fig. 5e,f). Neither application of Cho (n = 17) nor PC (n = 11) induced changes in currents of water injected control oocytes, which did neither express human α9 nor α10 nAChR subunits (Fig. 5g,h).

### PC interacts with heterologously expressed α9α10 nAChR

**Figure 6:**
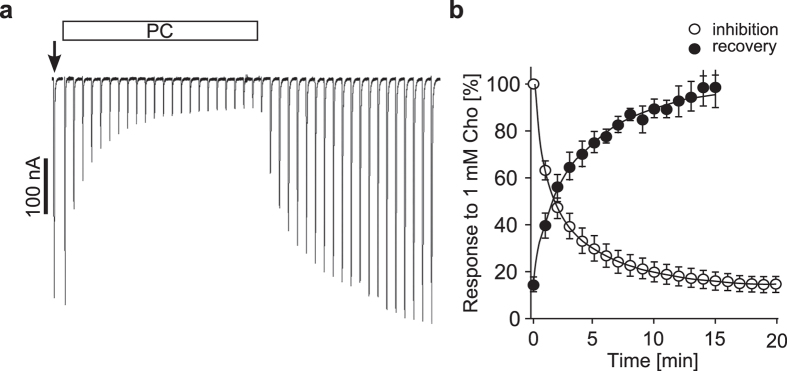
Phosphocholine inhibits choline-gated currents mediated by α9α10 nicotinic acetylcholine receptors heterologously expressed by Xenopus laevis oocytes. Two-electrode voltage-clamp experiments were performed on oocytes that heterologously expressed human α9α10 nicotinic acetylcholine receptors as described in Methods. (**a**) Representative current traces showing the inhibitory effects of phosphocholine (PC, 1 mM) on choline-gated currents (Cho, 1 mM). The current traces are each from 30 s recordings and are shown concatenated (omitting the 30 s gap between each individually recorded trace). Oocytes were continuously perfused with control solution and stimulated with 1 sec pulses of Cho once per min until steady-state baseline responses were observed (indicated by the arrow). Subsequently, the control solution was changed to one containing PC and the Cho-gated currents monitored for changes in amplitude for 20 min. Thereafter, PC was washed out and recovery from inhibition by PC monitored. (**b**) Analysis of inhibition and recovery from inhibition of Cho-gated currents by PC. The error bars denote the standard error of mean (SEM) from 5 oocytes.

Since PC did not evoke ion currents in oocytes expressing homomeric α9 and heteromeric α9α10 nAChR, we questioned whether PC might function as a silent desensitiser or as an antagonist of α9* nAChR containing subtypes. Therefore, we performed an additional set of experiments designed to monitor the effects of PC on the Cho-evoked responses over time (Fig. 6a,b). Oocytes expressing heteromeric α9α10 nAChR were stimulated with Cho once per min until a steady-state baseline was achieved. Then, the perfusion solution was switched to one containing 1 mM PC and the Cho-evoked responses were monitored for changes in amplitude. Under these conditions, PC decreased the Cho-evoked responses to 13.1 ± 3.3% (n = 5) of control values after a 20 min perfusion (Fig. 6b). PC was then washed out and Cho-evoked responses were monitored for recovery from inhibition. The responses recovered to 98.2 ± 8.2% (n = 5) of control values after a 15 min perfusion with control solution.

## Discussion

In the present study, we identify PC as a novel agonist of monocytic nAChR containing subunits α9 and α10. We provide evidence that α9 and α10 nAChR subunits are essential for the PC-mediated inhibition of ATP-induced ion channel functions at P2X7 receptor in monocytic cells and hence, for the inhibition of ATP-induced release of IL-1β. Cho, a well-known agonist of nAChR containing α9 subunits^21^, and PC provoke similar metabotropic but no ionotropic effects in monocytic cells. As expected, Cho induces ion current responses at conventional ionotropic α9 nAChR homomers or α9α10 heteromers. In stark contrast, PC does not provoke any ion current changes at these canonical ionotropic receptors. To the best of our knowledge, we are the first to describe an agonist of nAChR containing α9 subunits that triggers metabotropic but no ionotropic receptor functions.

In the first part of this study, we demonstrated that PC and Cho are agonists of metabotropic nAChR composed of α9 and α10 subunits in monocytic cells. We showed previously that PC and Cho dose-dependently inhibit the ATP-induced release of IL-1β from LPS-primed human monocytic U937 cells via nAChR that are sensitive to Mec, α-bungarotoxin and strychnine^20^. Here, we clarified that PC and Cho act as ligands of monocytic α9* nAChR by using the potent and selective antagonist RgIA4 which dose-dependently antagonized the inhibition of IL-1β release. These findings were further confirmed by gene-silencing experiments in U937 cells: silencing of *Chrna9* expression blunted the inhibitory effects of PC and Cho. The same observation was made, when *Chrna10* gene expression was silenced, suggesting that α9 and α10 nAChR subunits cooperate in monocytic cells.

The results of the gene-silencing experiments suggested but did not prove that α9 and α10 nAChR subunits are essential for the signalling of PC and Cho in monocytes. Therefore to corroborate the role of α9 nAChR subunit by an independent approach, we investigated freshly isolated PBMC from wild-type and gene-deficient mice. Although primary mouse PBMC were not intentionally primed to induce biosynthesis of IL-1β, they consistently released IL-1β in response to stimulation with BzATP. These observations are in accordance with previous findings on primary human PBMC^20^,^25^. We assume that freshly isolated PBMC became activated during cell isolation and culture^20^. As expected, PC and Cho efficiently inhibited the BzATP-induced release of IL-1β by PBMC obtained from wild-type mice but did not impair the IL-1β release from PBMC of *Chrna9* −/− and *Chrna10* −/− mice. From these data, we conclude that α9 and α10 subunits are essential for signalling of PC and Cho. Interactions of the α9 and α10 nAChR subunits were previously described. Subunits α10 do not assemble into functional ionotropic homomeric nAChR^26^, while co-expression of α9 and α10 nAChR subunits results in formation of functional heteromeric α9α10 nAChR^26^,^27^,^28^. Transcripts of α9 and α10 nAChR subunits have been detected in the auditory system^26^,^29^, dorsal root ganglion^30^, skin^31^, as well as in mononuclear leukocytes^20^,^32^,^33^ including human monocytic cells^20^.

Next, we confirmed our hypothesis that PC and Cho inhibit ATP-mediated ion current responses in monocytic cells. In accordance with our previous study^20^, we detected BzATP-induced ion current responses in whole-cell patch-clamp measurements on LPS-primed U937 cells. These currents are most likely due to activation of P2X7 receptors, as BzATP is a specific agonist of this ATP-receptor^34^, and due to a consecutive opening of pannexin hemichannels^35^,^36^. In line with our hypothesis, BzATP-mediated P2X7 receptor activation was completely abolished in presence of PC and Cho. This inhibitory effect was antagonized by the general nicotinic antagonist Mec and the α9*-specific conotoxin RgIA4. These results corroborate the involvement of α9 and/or α10 nAChR subunits in signalling of PC. A functional interaction of other subtypes of nAChR and P2X receptors was demonstrated previously in neurons^37^ and in heterologous expression systems^38^,^39^. In these studies, co-application of ATP and nicotinic agonists evoked current responses that were smaller than the sum of the individual currents induced by ATP and ACh or nicotine^37^,^38^. In the present study, PC or Cho alone did not evoke any ion current responses in U937 cells, consistent with functional coupling of a non-canonical metabotropic nAChR to ionotropic P2X7 receptors. While in excitable cells such as neurons nAChR are ligand-gated ion channels, no ionotropic nAChR functions have been observed in leukocytes^20^,^32^,^33^,^40^. At present, we do not know how activation of monocytic nAChR by PC or Cho translates into the observed inhibition of P2X7 receptor function.

In the last part of the study, we investigated the effect of PC at canonical ionotropic nAChR using *Xenopus laevis* oocytes as a heterologous expression system for human homomeric α9 nAChR as well as heteromeric α9α10 nAChR. Cho was included in these experiments as a positive control and transmembrane ion currents were recorded in TEVC measurements. We detected Cho-induced current responses in oocytes expressing homomeric α9 and heteromeric α9α10 nAChR. Current responses to the first Cho application for 30 s varied from 50 nA to 1200 nA (see Fig. 5a,f). Subsequent application of Cho resulted in smaller current responses, indicating receptor desensitisation. This is in contrast to previous studies and to our own observations, where application of ACh or Cho in short 1 s pulses resulted in repeatable current responses without desensitisation^27^,^41^. We assume that receptor desensitisation is a consequence of extended exposure of nAChR to Cho.

In sharp contrast to Cho, PC did not evoke ion current responses in α9 and/or α9α10 nAChR expressing oocytes. We conclude that at least in our experimental settings, PC is an agonist of metabotropic nAChR containing α9* subunits, whereas it does not stimulate canonical ligand-gated nAChR functions. This finding suggests that PC acts as a potent regulator of innate immunity but does not activate current responses in neuronal or non-neuronal cells expressing canonical ionotropic nAChR containing α9 subunits. Hence, PC and other molecules containing a PC group may be promising therapeutics for the prevention and treatment of excessive inflammation involving IL-1β such as life-threatening SIRS, without entailing the risk of adverse effects involving excitable cells.

Although PC did not trigger ion channel functions at α9 and α9α10 subunit containing nAChR expressed by *Xenopus laevis* oocytes, we obtained evidence that PC interacts with these canonical receptors and hypothesised that PC might act as a silent agonist. Per definition, silent agonists desensitise receptors without activating their function^42^,^43^. To further asses this, we used α9α10 nAChR expressing oocytes in an experimental setup that enabled application of Cho in 1 s pulses every 60 s, where Cho induced repetitive current responses without receptor desensitisation. We found that responses to Cho at heteromeric α9α10 nAChR were blunted in the presence of PC. The observed slow kinetics of inhibition and recovery from inhibition by PC was consistent with silent desensitisation. However, we should point out that our results do not rule out that the observed inhibition by PC is due to simple antagonism. Nicotine is an example of a ligand, which acts as an antagonist at ionotropic receptors^44^ containing α9 subunits, but acts as an agonist at monocytic metabotropic α9 nAChR that inhibit P2X7 function^20^. A silent agonist for homomeric nAChR was recently reported for homomeric α7 nAChR^43^. These authors showed that ACh-induced current responses were reduced after preincubation of the oocytes with compound NS6740^43^.

In conclusion, we identified PC as a novel agonist of metabotropic nAChR containing α9 and α10 subunits. PC and Cho evoke no ion current responses at these receptors expressed by monocytes but efficiently inhibit ATP-mediated P2X7 receptor activation and release of IL-1β. In contrast to Cho, PC does not trigger ionotropic functions at canonical human α9 nAChR homomers and α9α10 nAChR heteromers. These findings suggest that PC may be a valuable active substance for the treatment of inflammatory diseases that targets nAChR of monocytes without disturbing ionotropic functions of excitable cells.

## Methods

### U937 cell culture and stimulation

U937 cells were obtained from the German Collection of Microorganisms and Cell Cultures (Braunschweig, Germany). The cells were cultured in RPMI 1640 (Gibco by Life Technologies GmbH, Darmstadt, Germany) supplemented with 10% fetal calf serum (FCS; Biochrome, Berlin, Germany) and 2 mM L-glutamine (Gibco by Life Technologies GmbH) under 5% CO_2_ atmosphere at 37 °C.

To investigate IL-1β release cells were transferred to 24-well plates (1 × 10^6^ cells/ml and per well). Cells were primed with 1 μg/ml LPS from *Escherichia coli* (L2654; 1 μg/ml; Sigma-Aldrich, Deisenhofen, Germany) for 5 h. After priming, the P2X7 receptor agonist BzATP (Sigma-Aldrich; 100 μM) was added for 30 min in presence or absence of different concentrations of cholinergic agonists and antagonists. Cho chloride (100 μM), PC chloride calcium salt tetrahydrate (100 μM), and Mec hydrochloride (100 μM) were purchased from Sigma-Aldrich. An analogue of α-conotoxin RgIA (RgIA4)^22^ was used in concentrations from 0.2 to 200 nM. After cell treatment, cells were spun down (500 g, 8 min) the supernatants were collected and stored at −20 °C. IL-1β concentrations were measured using a human Quantikine Immunoassays (R&D Systems, Minneapolis, MN) and LDH was determined.

### Silencing of α9 and α10 nAChR subunit expression

In some experiments the expression of nAChR containing the α9 and/or α10 subunits in U937 cells was reduced by using siRNA technology. U937 cells were transfected with ON-TARGETplus human *Chrna9* or *Chrna10* siRNA SMARTpool (Thermo Fisher Scientific, Schwerte, Germany). As a control for unspecific effects of siRNA transfection cells were transfected with negative control ON-TARGETplus Non-targeting Control Pool (Thermo Fisher Scientific). In accordance with the manufacturer’s protocol, all cells were transfected with 30 pM siRNA/1 × 10^6^ cells using Amaxa Cell Line Nucleofector Kit C and Nucleofector II Device (both from Lonza Cologne, Cologne, Germany). 48 h after siRNA transfection, IL-1β release experiments were performed.

### Mononuclear leukocytes from *Chrna9* and *Chrna10* gene-deficient mice

Male and female gene-deficient *Chrna9* (129S-*Chrna9*^tm1Bedv^/J)^45^ and *Chrna10* (129S4-*Chrna10*^tm1Bedv^/Mmucd)^46^ as well as corresponding WT mice (for details see^45^,^46^) were used for isolation of PBMC. Experimental animals received humane care according to NIH “Guide for the Care and Use of Laboratory Animals”. Animal experiments were approved by the local committee at the Regierungspräsidium Giessen, Hesse, Germany (permit No. Gi 20/23-A10/2011).

Mice were euthanized by neck dislocation, and blood was drawn from the caval vein into heparinized syringes. PBMC were separated by discontinuous Percoll (Ge Healthcare Bio-Sciences AB, Uppsala, Sweden; 1.082 g/ml) density gradient centrifugation and cultured for 2 h in RPMI 1640 (Gibco by Life Technologies GmbH) supplemented with 10% FCS (Biochrome) and 2 mM L-glutamine (Gibco by Life Technologies GmbH), at 5% CO_2_ and 37 °C. For investigation of IL-1β release, BzATP (Sigma-Aldrich; 100 μM) was added for 30 min in the presence or absence of PC (100 μM) or Cho (100 μM). Subsequently, cell culture supernatants were collected and stored at −20 °C. Finally, IL-1β concentrations were measured by using mouse Quantikine IL-1β Immunoassay (R&D Systems).

### LDH measurements

In order to test for cell viability, activity of the cytoplasmic enzyme LDH was assayed by the Non-Radioactive Cytotoxicity Assay (Promega, Madison, WI) according to the supplier’s instructions. LDH in the cell culture supernatants of U937 cells and PBMC was measured at the end of the experiments. For calculating the proportion of dead cells, a maximum LDH release control was generated. For this purpose, U937 cells were lysed by freezing them twice (−80 °C). Subsequently, the samples were analysed according to the supplier’s instructions and values determined in cell culture supernatants were compared with the total content of LDH in lysed cells.

### Whole-cell patch-clamp recordings on U937 cells

For electrophysiological recordings, U937 cells were placed in poly-L-lysine coated cell culture dishes (Nunc, Roskilde, Denmark) containing bath solution (in mM: 5.4 KCl, 120 NaCl, 2 CaCl_2_, 1 MgCl_2_, 25 glucose and 10 HEPES (4-(2-hydroxyethyl)-piperazine-1-ethanesulfonic acid); pH 7.4).

Patch pipettes with a resistance of 2–3 MΩ were pulled from borosilicate glass capillaries (Hilgenberg, Malsfeld, Germany) using a dmz-puller (Zeitz, Augsburg, Germany) and filled with a pipette solution (in mM: 120 KCl, 1 CaCl_2_, 2 MgCl_2_, 10 HEPES, 11 EGTA (ethylene glycol tetraacetic acid), and 20 glucose; pH 7.3). After 5 h incubation with LPS, whole-cell patch-clamp recordings were performed at room temperature. Cells were clamped to −60 mV and transmembrane currents were recorded using an EPC-9 amplifier (HEKA, Lambrecht, Germany) and acquired via an ITC-16 interface with Pulse software (HEKA). BzATP (100 μM), PC (1 mM), Cho (100 μM), RgIA4 (200 nM) and Mec (100 μM) were dissolved in bath solution and applied via a pressure-driven microperfusion system. Rg1A4 (sequence ID3) was prepared as previously described^22^. All chemicals used for preparation of bath and pipette solution were purchased from Fluka (Deisenhofen, Germany), except for HEPES and EGTA (Sigma-Aldrich).

At least once per measuring day respective control experiments were performed in which BzATP was applied twice and the BzATP-induced effect was tested for reversibility and repeatability.

### Heterologous expression of human nAChR in oocytes and TEVC measurements

Oocytes were obtained from adult female South African Clawed Frogs (*Xenopus laevis*; *Xenopus*-Express, Le Bourg, France). Manipulations of animals were conducted in accordance to the guidelines of the German law of animal care and were authorized by the local committee at the Regierungspräsidium Giessen, Hesse, Germany (permit number 400_M and 478_M). Oocytes were separated by collagenase treatment (1.5 mg/ml; Biochem, Karlsruhe, Germany) for 90 min. The follicle layer was removed by incubation of cells in Ca^2+^-free oocyte Ringer’s solution (ORi) containing (in mM): 90 NaCl, 1 KCl, 5 HEPES, and 1 EGTA (pH 7.4) for 10 min. Defolliculated oocytes were stored in a low-Na^+^ solution (in mM): 10 NaCl, 80 NMDG–Cl (N-methyl-d-glucamine), 1 KCl, 2 CaCl_2_, 5 HEPES, 2.5 Na-pyruvate (Applichem, Darmstadt, Germany), 0.06 penicillin (Sigma-Aldrich), 0.02 streptomycin (Sigma-Aldrich) at 17 °C (pH 7.4).

Plasmid DNA encoding the human *Chrna9*, human *Chrna10* as well as the human 43 kDa receptor-associated protein of the synapse (*RAPSN*) were obtained from Eurofins Genomics (Ebersberg, Germany). Capped cRNA was synthesized using an *in vitro* transcription kit (T7-RiboMAX™ Large Scale RNA Production System Kit, PROMEGA, Mannheim, Germany).

Oocytes of stages V and VI (Dumont 1972) were injected with cRNA encoding α9 nAChR subunits (20 ng per oocyte) or α9α10 nAChR subunits (each 20 ng per oocyte) using a microinjector (Nanoject, Drummond Scientific, Broomall, USA). In order to increase expression levels and obtain stable nAChR expression, cRNA encoding *RAPSN* (5 ng per oocyte) was co-injected in both cases^47^,^48^. All cRNA was dissolved in nuclease-free water. The injection volume was 50.6 nl. In all TEVC experiments representative controls were performed with oocytes that were injected with 50.6 nl of nuclease-free water.

After an incubation time of 3–5 days, the transmembrane currents (I_M_) of water- or RNA-injected oocytes were recorded by the TEVC technique. Oocytes were placed in a perfusion chamber and perfused (gravity driven) with ORi containing (in mM): 90 NaCl, 1 KCl, 2 CaCl_2_, and 5 HEPES (pH 7.4). Intracellular microelectrodes were pulled from borosilicate glass capillaries and filled with 1 M KCl solution. The membrane voltage was clamped to −60 mV using a TEVC amplifier (Warner Instruments, Hamden, USA), and transmembrane currents were low-pass filtered at 1000 Hertz (Frequency Devices 902, Haverhill, Massachusetts, USA) and recorded with a strip chart recorder (Kipp & Zonen, Delft, The Netherlands). In all experimental groups, measurements were performed on oocytes from at least two different *Xenopus laevis* individuals.

For experiments examining the effects of continuous exposure to PC on α9- and α10-mediated currents, oocytes were injected with a 1:1 ratio of cRNA for human α9 and α10 nAChR subunits and incubated at 17 °C for 3 days. To conduct TEVC experiments, the oocytes were placed in a 30 μl chamber and continuously perfused by gravity with a solution containing 96 mM NaCl, 2.5 mM KCl, 1.8 mM CaCl_2_, and 1 mM MgCl_2._ The pH of the solution was adjusted to 7.4 with NaOH. A stock solution of 100 mM PC was prepared in distilled water. A working solution of 1 mM PC was prepared in perfusion solution containing lower CaCl_2_ (0.8 mM) such that the final concentration of Ca^2+^ in all solutions was 1.8 mM. The oocyte membranes were clamped at a holding potential of −70 mV and stimulated with 1 s pulses of 1 mM Cho once every 60 s until a steady-state baseline response was observed. The perfusion solution was then switched on one containing 1 mM PC and the oocytes stimulated with 1 s pulses of 1 mM Cho plus 1 mM PC and the Cho-evoked responses monitored for changes in amplitude. The data for inhibition of the Cho-evoked responses were normalized to 3 averaged control pulses and analysed with an exponential decay equation. Data for recovery from inhibition were analysed with an exponential association equation. The data for inhibition by PC were best fit with a double exponential and the data for recovery from inhibition with a single exponential.

### Statistical analyses

Data were analysed with the SPSS software (Munich, Germany) or GraphPad Prism 6 software (Ja Lolla, CA, USA). Values derived from different cells were compared, where applicable, by the nonparametric Kruskal-Wallis test, followed by the Mann-Whitney rank-sum test. The Wilcoxon signed-rank test was used for analyses of dependent values. The number (n) of individual experiments is indicated in the Results section and the Figures. In TEVC measurements oocytes from at least two different *Xenopus laevis* individuals were used.

## Additional Information

**How to cite this article**: Richter, K. *et al*. Phosphocholine – an agonist of metabotropic but not of ionotropic functions of α9-containing nicotinic acetylcholine receptors. *Sci. Rep.* **6**, 28660; doi: 10.1038/srep28660 (2016).

## Figures and Tables

**Table 1:** Lactate dehydrogenase (LDH) release of U937 cells.

Cell treatment	Cell death [%] Mean ± SEM	n
–	2.14 ± 0.87	6
LPS	2.23 ± 0.91	6
LPS, BzATP	1.65 ± 0.67	6
LPS, BzATP, PC	1.76 ± 0.72	6
LPS, BzATP, PC, RgIA4 200 nM	2.38 ± 0.97	6
LPS, BzATP, PC, RgIA4 20 nM	1.48 ± 0.60	6
LPS, BzATP, PC, RgIA4 2 nM	2.30 ± 0.94	6
LPS, BzATP, PC, RgIA4 0.2 nM	1.76 ± 0.72	6
LPS, BzATP, Cho	1.30 ± 0.53	6
LPS, BzATP, Cho, RgIA4 200 nM	1.50 ± 0.61	6
LPS, BzATP, Cho, RgIA4 20 nM	1.54 ± 0.63	6
LPS, BzATP, Cho, RgIA4 2 nM	1.66 ± 0.68	6
LPS, BzATP, Cho, RgIA4 0.2 M	2.09 ± 0.85	6
si control: LPS	4.96 ± 2.48	4
si control: LPS, BzATP	5.04 ± 2.52	4
si control: LPS, BzATP, PC	5.41 ± 2.71	4
si control: LPS, BzATP, Cho	5.13 ± 2.57	4
si *Chrna9*: LPS	4.55 ± 2.27	4
si *Chrna9*: LPS, BzATP	5.20 ± 2.60	4
si *Chrna9*: LPS, BzATP, PC	5.13 ± 2.56	4
si *Chrna9*: LPS, BzATP, Cho	5.00 ± 2.50	4
si *Chrna10*: LPS	2.48 ± 1.01	6
si *Chrna10*: LPS, BzATP	3.25 ± 1.33	6
si *Chrna10*: LPS, BzATP, PC	3.00 ± 1.22	6
si *Chrna10*: LPS, BzATP, Cho	2.96 ± 1.21	6

Human monocytic U937 cells were primed for 5 h with lipopolysaccharide (LPS, from *Escherichia coli*; 1 μg/ml). Subsequent, BzATP (100 μM) was applied for another 30 min in presence or absence of the nAChR agonist phosphocholine (PC; 100 μM) or choline (Cho; 100 μM) or α-conotoxin RgIA4. In some experiments the expression of nAChR containing the α9 or α10 subunit was reduced by using the small interfering RNA (si) transfection. At the end of the experiments, LDH was measured in the cell culture supernatants and is given as % of total release (mean ± standard error of mean, SEM).

**Table 2:** Lactate dehydrogenase (LDH) measurement of peripheral blood mononuclear leukocytes (PBMC).

Genotype	Cell treatment	Cell death [%] Mean ± SEM	n
*Chrna9* +/+	–	6.90 ± 1.50	6
*Chrna9* +/+	BzATP	6.97 ± 1.37	6
*Chrna9* +/+	BzATP, PC	6.62 ± 1.06	6
*Chrna9* +/+	BzATP, Cho	6.71 ± 1.23	6
*Chrna9* −/−	–	8.64 ± 0.93	5
*Chrna9* −/−	BzATP	7.63 ± 1.09	5
*Chrna9* −/−	BzATP, PC	7.96 ± 0.91	5
*Chrna9* −/−	BzATP, Cho	8.62 ± 1.06	5
*Chrna10* +/+	–	4.38 ± 0.95	4
*Chrna10* +/+	BzATP	5.04 ± 2.04	4
*Chrna10* +/+	BzATP, PC	4.81 ± 1.96	4
*Chrna10* +/+	BzATP, Cho	5.96 ± 1.89	4
*Chrna10* −/−	–	6.35 ± 1.18	6
*Chrna10* −/−	BzATP	6.42 ± 1.13	6
*Chrna10* −/−	BzATP, PC	6.40 ± 1.30	6
*Chrna10* −/−	BzATP, Cho	7.68 ± 1.38	5

Peripheral blood mononuclear leukocytes (PBMC) were isolated from *Chrna9* gene-deficient (*Chrna9* −/−) and *Chrna10* (*Chrna10* −/−) gene-deficient mice as well as from two corresponding wild-type (WT) strains. LDH was measured in the cell culture supernatants at the end of the experiments, and is given as % of total release (mean ± standard error of mean, SEM). Untreated cells are marked by “–”. BzATP (100 μM) was applied in the presence and absence of phosphocholine (PC; 100 μM) or choline (Cho; 100 μM).
